# Rates of, and risk factors for, septic arthritis in patients with invasive pneumococcal disease: prospective cohort study

**DOI:** 10.1186/s12879-017-2797-7

**Published:** 2017-10-12

**Authors:** Thomas J. Marrie, Gregory J. Tyrrell, Sumit R. Majumdar, Dean T. Eurich

**Affiliations:** 1 Department of Medicine, Dalhousie University, Halifax, NS Canada; 2 Division of Diagnostic and Applied Microbiology, Department of Laboratory Medicine and Pathology, University of Alberta, Edmonton, AB Canada; 3 The Provincial Laboratory for Public Health, Edmonton, AB Canada; 4 Department of Medicine, Faculty of Medicine and Dentistry, University of Alberta, Edmonton, AB Canada; 5 School of Public Health, 2-040 Li Ka Shing Center for Health Research Innovation, University of Alberta, Edmonton, AB Canada

**Keywords:** Septic arthritis, *Streptococcus Pneumoniae*, Risk factors

## Abstract

**Background:**

There are many case reports of septic arthritis complicating invasive pneumococcal disease (IPD); however, no study has compared patients with IPD with septic arthritis to those who didn’t develop septic arthritis Thus, we aimed to determine the rates of, and risk factors for, septic arthritis in patients with invasive pneumococcal disease (IPD).

**Methods:**

Socio-demographic, clinical, and serological data were captured on all patients with IPD in Northern Alberta, Canada from 2000 to 2014. Septic arthritis was identified by attending physicians. Descriptive statistics and multivariate analyses were used to compare characteristics of those with septic arthritis and IPD to those who did not.

**Results:**

Septic arthritis developed in 51 of 3251 (1.6%) of patients with IPD. Inability to walk independently, male sex, and underlying joint disease were risk factors for developing septic arthritis in patients with IPD. Capsular serotypes 22 and 12F were more common in patients with septic arthritis than those without.

**Conclusions:**

In patients with IPD, septic arthritis is uncommon. Certain risk factors such as walking with or without assistance and underlying joint disease make biological sense as damaged joints are more likely to be infected in the presence of bacteremia.

**Trial registration:**

Not applicable.

## Background

*Streptococcus pneumoniae* is thought to account for about 3-5% of all cases of septic arthritis [1]. There are many case reports of this entity [2] and there have been several reviews, the most comprehensive of which examined the features of 190 cases (108 adults) reported between 1965 and 2003 [3]. While the clinical features of this entity have been adequately described there has been no study that has compared patients with invasive pneumococcal disease (IPD) with septic arthritis to those who didn’t develop septic arthritis. Such a comparison would allow a delineation of the risk factors for septic arthritis. A 15-year study of IPD in Northern Alberta gave us the opportunity to do this. In addition to examining rates of, and risk factors for, septic arthritis we hypothesized that patients with septic arthritis would have more severe infection as manifested by intensive care admission, higher complication rate and higher mortality rate.

## Methods

### Definitions

Cases of IPD were defined as per the national case definition of isolation of *S. pneumoniae* from a normally sterile site such as blood, CSF, pleural fluid, biopsy tissue, joint aspiration, pericardial fluid, or peritoneal fluid [4]. IPD is a provincially notifiable disease in Alberta therefore all invasive pneumococcal isolates are submitted to the Provincial Laboratory for Public Health (PLPH) for further characterization. This allowed us to prospectively identify all cases of IPD in Northern Alberta. Septic arthritis was a diagnosis made by the attending clinicians based on clinical findings and aspiration of joint fluid. All patients without a diagnosis of septic arthritis were considered as not having the disease.

### Clinical data collection

Research nurses collected sociodemographic, clinical, functional, and laboratory data using a standardized case report form (CRF). The research nurses received training on data collection prior to the start of the study. In addition to the CRF, standard operating procedures documents, definitions, drug classification and underlying illness categorization were part of their working documents. With respect to underlying illnesses, if the attending physician recorded such an illness it was accepted as such for the purpose of the study. If there was no recording of an underlying illness, it was assumed that the illness was not present. Complete data capture was available for sociodemographic and functional status. Approximately 2% of patients were missing laboratory values (hemoglobin, white blood cell count, or platelets) from the no septic arthritis group exclusively and were otherwise excluded from analyses specific to those endpoints. This study received approval from the institutional research review committees of the Alberta Health Regions as well as the University of Alberta ethics review board.

### Identification and Serotyping of S. Pneumoniae isolates

*S. pneumoniae* isolates were received at the PLPH from acute diagnostic laboratories in Alberta as per requirements of provincial notifiable disease regulations. *S. pneumoniae* isolates were confirmed as *S. pneumoniae* based on characteristic morphology and optochin susceptibility prior to serotyping [5]. All pneumococcal isolates that exhibited a positive Quellung reaction using commercial type specific antisera obtained from Statens Serum Institute, Copenhagen, Denmark were assigned a serotype designation [6] Strains that were susceptible to optochin but which failed to serotype using the Quellung assay, were assayed further using AccuProbe™ *Streptococcus pneumoniae* culture identification test, Gen-Probe, San Diego, CA, to confirm the species identification.

### Statistical analysis

Sociodemographics, clinical characteristics, and major in-hospital complications (e.g., presence of cellulitis, meningitis, admission to the intensive care unit (ICU), all-cause in-hospital mortality) are presented using standard descriptive statistics for those with and without septic arthritis using chi-squared tests or Fisher’s exact tests for categorical data, where appropriate, and Student’s t-test for continuous data. We also evaluated the independent effects of characteristics associated with septic arthritis using a backward stepwise multivariate logistic regression analyses (*p* < 0.1 to remain in the model) for bivariate data and a similar linear regression approach for continuous data. In addition, IPD serotypes were explored between the groups. All analyses were performed with Stata SE, version 12.1 (Stata, College Station, TX).

## Results

Over the course of the study 3251 patients had IPD of whom 51 (1.6%) had septic arthritis. The patients with septic arthritis were less likley to walk independently and less likely to have chronic obstructive pulmonary disease (Table 1). 27.4% of the septic arthritis patients had underlying joint disease compared with 8.5% in the non-septic arthritis group (Table 1). In 9 (18%) patient’s joint involvement was polyarticular. The knee was the most frequently infected joint – 26 (51%), followed by shoulder 7 (13.7%); elbow 6 (11.7%); wrist 6 (11.7%), hip 4 (7.8%); ankle 4 (7.8%) and acromioclavicular joint 1 (1.9%). In multivariate analyses, septic arthritis patients were more likely to have osteoarthritis (adjusted odds ratio (aOR) 3.4, 95% confidence interval (95%CI) 1.55-7.34) and rheumatoid arthritis (aOR 5.05, 95%CI 1.85-13.78), more likely to be males (aOR 2.07, 95%CI 1.12-3.83), lesslikely to walk independently (aOR 0.48, 95%CI 0.24-0.96) and less likely to have chronic obstructive pulmonary disease (aOR 0.14, 95%CI 0.03-0.57). No other characteristics were associated with septic arthritis (*p* > 0.1).

**Table 1:** Demographic features of those with invasive pneumococcal disease and septic arthritis and those with IPD but without septic arthritis

	Septic Arthritis	No Septic Arthritis	Unadjusted *P*-value
Number	51 (1.6)	3200 (98.4)	
Male	35 (68.6)	1803 (56.3)	0.08
Mean age (SD)	56.3 (17)	54.6 (17.4)	0.44
Aboriginal	9 (17.6)	421 (13.2)	0.35
Homeless	2 (4)	232 (7.3)	0.12
Current tobacco smoker	23 (45.1)	1444 (45.1)	0.99
Illicit drug use	9 (17.6)	550 (17.2)	0.93
Alcoholism	14 (27.5)	771 (24.1)	0.58
Functional status in week prior to hospital admission			
Walking without assistance	38 (74.5)	2738 (85.6)	0.027
Co-morbid illness those with invasive pneumococcal disease and septic arthritis and those with IPD but without septic arthritis			
Any Comorbidity	48 (94.1)	2738 (85.6)	0.83
Cancer in past 5 years	5 (9.8)	399 (12.5)	0.57
Solid organ transplant	0	18 (0.6)	1.00
Bone Marrow Transplant	1 (2)	36 (1.1)	0.45
Rheumatoid arthritis	5 (9.8)	73 (2.3)	0.007
Osteoarthritis	9 (17.6)	206 (6.4)	0.001
Gout	1 (2)	28 (0.8)	0.37
Pseudogout	1 (2)	1 (0.03)	0.03
Past myocardial infarction	1 (2)	203 (6.3)	0.37
Diabetes	3 (5.9)	193 (6.0)	0.97
HIV	0	136 (4.3)	0.27
COPD	2 (3.9)	562 (17.6)	0.01
Splenectomy	2 (3.9)	49 (1.5)	0.17

Table 2 shows that the septic arthritis group were less likely to be admitted to ICU which remained after adjustment for potential characteristics in Table 1 (aOR 0.41, 95%CI 0.18-0.92), *P* = 0.03) There was no difference in other measures of severity of illness such as mechanical ventilation, acute kidney injury, empyema, cellulitis, bacteremia in either univariate or adjusted analyses between those with and without septic arthritis. With respect to laboratory data, no differences were observed between those with and without septic arthritis in either univariate or multivariate analyses with the exception that septic arthritis patients tended to have higher platelet counts even after adjustment for potential characteristics in Table 1.

**Table 2:** Severity of illness and outcomes patients with invasive pneumococcal disease with and without septic arthritis

Outcomes	Septic Arthritis	No Septic Arthritis	Unadjusted *P*-value
Intensive Care Unit Admission	7 (13.7)	876 (27.4)	0.04
Mechanical ventilation	5 (9.8)	680 (21.3)	0.055
Meningitis	0 (0)	160 (5)	0.18
Bacteremia	51 (100)	3061 (96)	0.17
Empyema	5 (9.8)	215 (6.7)	0.81
Cellulitis	2 (3.9)	81 (2.5)	0.53
Creatinine increase of >100 umol/L	2 (3.9)	116 (3.6)	0.91
Hemoglobin g/L^a^	119.5 (25.0)	122.2 (19.6)	0.46
WBC × 10^9^ /L^b^	16.0 (1.0)	17.8 (0.4)	0.51
Platelets × 10^9^ /L^c^	251.3 (25.9)	213.6 (2.1)	0.03
In hospital mortality	6 (11.8)	480 (15)	0.52

^a^60 no septic arthritis patients missing data

^b^57 no septic arthritis patients missing data

^c^68 no septic arthritis patients missing data

Table 3 shows the five most common serotypes of *S. pneumoniae* causing infection in the septic arthritis group. Two of these serotypes, 22F and 12F were more frequent in the septic arthritis group than in those without septic arthritis.

**Table 3:** Top 5 serotypes causing invasive pneumococcal disease in patients with and without septic arthritis

Serotype	Septic Arthritis	No Septic Arthritis	Unadjusted *P*-value
22F	7 (13.7)	213 (6.7)	0.046
4	7 (13.7)	299 (9.3)	0.3
8	7 (13.7)	254 (7.9)	0.13
12F	5 (9.8)	98 (3.1)	0.006
5	3 (5.9)	274 (8.6)	0.50

## Discussion

This study found that septic arthritis was not that common (1.6% incidence in this cohort of IPD patients). Although this is the first study we are aware of that focused exclusively on rates of septic arthritis among patients with IPD, previous studies of septic arthritis patients have reported that pneumococcal septic arthritis accounts for 3-8% [1–3, 7] of all septic arthritis cases. Factors associated with septic arthritis in patients with IPD included underlying joint disease, male sex, and mobility.

Pre-existing joint disease has been documented in other studies as predisposing factors for septic arthritis [1–3]. Furthermore, we suspect that many of the patients who reported limitations in walking did so as a result of (osteo)arthritis which may not have been captured in the medical record. In keeping with other reports, we found that the knee is most the commonly involved joint and that polyarticular infection was not uncommon [1–3]. A damaged joint is more likely to become infected in patients with bacteremia than an undamaged joint. In a prospective study of 75 patients with septic arthritis 46 (61%) had underlying joint disease [8]. In our study (27.4%) of the patients with pneumococcal septic arthritis had underlying joint disease compared with 8.5% of those without septic arthritis. The finding that male sex is at increased risk is not consistent with previous studies where males and females appear to have similar risk or potentially a slight female predominance [1–3, 9].

Although previous studies have suggested meningitis may be a risk factor for septic arthritis [1, 7], among our IPD patients we observed the opposite with no patients having concurrent meningitis and septic arthritis. The reason why meningitis could be a risk factor for septic arthritis is not well understood. One possibility is that high bacterial load may be more likely to result in seeding of the joints and the meninges.

With respect to microorganism specific factors, while 4 of the 5 most common serotypes in septic arthritis patients were more frequently represented compared with non-septic arthritis patients none achieved statistical significance. Interestingly the two serotypes that were significantly higher in septic arthritis patients, 12 F and 22 F, are high case fatality rate serotypes [10]. Thus, whether or not there are organism specific factors remains to be determined.

A strength of this study is the large number of patients with IPD and extensive clinical data. A limitation of our study is that we did not measure bacterial load in patients with IPD. It is possible that a higher bacterial load results in seeding of the joints. In addition, we did not undertake detailed examinations of fluid and aspirates for alternate etiologies of inflammatory arthritis in this population of IPD patients, such as gout or pseudogout. Last, although patients reported limitations in ambulation prior to hospitalization, we do not know to what degree this is simply a proxy measure for undiagnosed joint disease or perhaps the presence of a prosthetic joint.

## Conclusion

In conclusion, in patients with IPD, septic arthritis is uncommon. Certain risk factors such as inability to walk independently without assistance and underlying joint disease make biological sense as damaged joints are more likely to be infected in the presence of bacteremia.
